# Distribution of a Sulfolane-Metabolizing *Rhodoferax* sp. Throughout a Contaminated Subarctic Aquifer and Two Groundwater Treatment Systems

**DOI:** 10.3389/fmicb.2021.714769

**Published:** 2021-08-26

**Authors:** Christopher P. Kasanke, Michael D. Willis, Mary Beth Leigh

**Affiliations:** Institute of Arctic Biology, University of Alaska Fairbanks, Fairbanks, AK, United States

**Keywords:** *Rhodoferax*, emerging contaminant, granular activated carbon, aerobic biodegradation, air sparging

## Abstract

An extensive plume of the emerging contaminant sulfolane has been found emanating from a refinery in Interior Alaska, raising questions about the microbial potential for natural attenuation and bioremediation in this subarctic aquifer. Previously, an aerobic sulfolane-assimilating *Rhodoferax* sp. was identified from the aquifer using stable isotope probing. Here, we assessed the distribution of known sulfolane-assimilating bacteria throughout the contaminated subarctic aquifer using 16S-rRNA-amplicon analyses of ~100 samples collected from groundwater monitoring wells and two groundwater treatment systems. One treatment system was an *in situ* air sparging system where air was injected directly into the aquifer. The other was an *ex situ* granular activated carbon (GAC) filtration system for the treatment of private well water. We found that the sulfolane-assimilating *Rhodoferax* sp. was present throughout the aquifer but was significantly more abundant in groundwater associated with the air sparge system. The reduction of sulfolane concentrations combined with the apparent enrichment of sulfolane degraders in the air sparging zone suggests that the addition of oxygen facilitated sulfolane biodegradation. To investigate other environmental controls on *Rhodoferax* populations, we also examined correlations between groundwater geochemical parameters and the relative abundance of the *Rhodoferax* sp. and found only manganese to be significantly positively correlated. The sulfolane-assimilating *Rhodoferax* sp. was not a major component of the GAC filtration system, suggesting that biodegradation is not an important contributor to sulfolane removal in these systems. We conclude that air sparging is a promising approach for enhancing the abundance and activity of aerobic sulfolane-degraders like *Rhodoferax* to locally stimulate sulfolane biodegradation *in situ*.

## Introduction

Sulfolane is an anthropogenic organosulfur compound used in industrial applications, worldwide (Tindal et al., 2006). Developed in the 1950s, sulfolane meets the criteria for classification as a contaminant of emerging concern due to its persistence, mobility, continued widespread use, and lack of inclusion in routine contaminant assessments (Lapworth et al., 2012; Sauve and Desrosiers, 2014). An environmentally and economically notable case of sulfolane contamination occurred in North Pole, Alaska, where sulfolane released from a petroleum refinery has contaminated the surrounding groundwater. The contaminant plume is 5.6km long, 3.2km wide, and over 90m deep, affecting hundreds of residential drinking water wells (Magdziuk and Andresen, 2018). The effects of sulfolane exposure on humans are unknown, but a study that exposed rats to sulfolane *via* drinking water resulted in lowered white blood cell counts in females and neuropathy in males (Petersen et al., 2012), providing cause for human and environmental health concern. Yet, relatively little is known about the biodegradation of sulfolane, which limits the understanding of its fate and the potential for natural attenuation or bioremediation in contaminated environments.

Aerobic sulfolane biodegradation has been observed in several laboratory incubation studies using solids and/or groundwater from contaminated aquifers (Fedorak and Coy, 1996; Greene et al., 2000; Kasanke and Leigh, 2017). However, reports of biodegradation under anaerobic conditions that are more prevalent in the subsurface are rare and have inconsistent findings (Greene et al., 1998; Kim et al., 1999). Prior aerobic and anaerobic microcosm studies using groundwater and sediment from the sulfolane-contaminated aquifer in North Pole, Alaska revealed aerobic biodegradation to be the only observable mechanism of sulfolane loss (Kasanke and Leigh, 2017). This is consistent with the large size of the contaminant plume, indicating that no appreciable biodegradation has occurred *in situ*. As part of testing potential remediation solutions, *in situ* experimental air sparge (AS) system was employed that injected atmospheric air into a small section of the North Pole aquifer within the refinery property in an attempt to reduce off-site migration of sulfolane (Angermann and DeJournett, 2013). Air sparging was effective at lowering sulfolane concentrations; however, the mechanisms behind the reduction in contaminant concentrations were not conclusively determined (Kurapati et al., 2014). Given the rapid aerobic biodegradation observed in aquifer materials in our prior laboratory experiments, we hypothesized that air sparging stimulated the growth and activity of indigenous microbial populations capable of biodegrading sulfolane.

Residents with contaminated drinking water wells were provided with alternative drinking water or granular activated carbon (GAC) filters to remove the sulfolane from contaminated well water (Magdziuk et al., 2016) by refinery owners. While sorption was assumed to be the mode of action for sulfolane removal using GAC, the potential for biodegradation to occur within domestic treatment systems was of interest due to the potential to create degradative intermediates of unknown toxicity. Prior to home installation, the North Pole GAC point-of-entry water treatment systems installed to treat private wells were tested and found to be effective in sulfolane removal (BARR, 2011) likely because GAC rapidly sorbs sulfolane (Diaz, 2015). However, inoculation with an aerobic, sulfolane-degrading enrichment culture was necessary to effectively remediate sulfolane-contaminated groundwater in California (Ying et al., 1994). Ying et al. (1994) concluded that biodegradation, not sorption, was responsible for the effectiveness of their GAC system. Microcosm studies using GAC from a North Pole point-of-entry remediation system as inoculum found no biodegradation after 10weeks of incubation under aerobic conditions (Janda, 2016). However, the GAC is replaced in 6-month intervals and it is unknown if sulfolane biodegradation occurs during this more prolonged time period (BARR, 2011). Understanding the mechanisms underlying GAC treatment system function is necessary to fully assess their impact on human health.

Recently, DNA-based stable isotope probing identified a *Rhodoferax* sp. as the dominant sulfolane-metabolizing species in the North Pole aquifer microbial community (Kasanke et al., 2019). Other taxa of sulfolane-degrading bacteria have been identified in other locations, including a *Variovorax* sp. isolated from Alberta; Canada (Greene et al., 2000), *Pseudomonas maltophilia* isolated from Illinois; United States (Lee and Clark, 1993), a novel *Shinella* sp. isolated from Okinawa Main Island; Japan (Matsui et al., 2009), and most recently, a strain of *Cupriavidus plantarum* isolated from a petrochemical wastewater treatment plant (Yang et al., 2019). Although environmental microorganisms that can degrade sulfolane have been identified, the abundance and distribution of these microbes throughout a contaminated aquifer and the environmental parameters that control their abundance have not been assessed. Characterizing the distribution of sulfolane-degrading microbes throughout a contaminated aquifer and remediation systems informs plume longevity estimates, identifies potential areas of active intrinsic biodegradation, and may provide insight into the fundamental mechanisms underlying effective biostimulation systems such as air sparging.

The objective of this study was to assess the distribution of sulfolane-degrading bacteria in a contaminated aquifer and in two different remediation systems (air-sparging and GAC) in order to understand the mechanisms and controls on sulfolane biodegradation. We examined the microbial communities in 100 groundwater monitoring wells (MW) distributed throughout the North Pole aquifer including an *in situ* air sparging system for the relative abundance and distribution of a recently identified sulfolane-metabolizing *Rhodoferax* sp. as well as other known sulfolane degraders. In an effort to identify environmental controls on biodegradation potential, we examined correlations between environmental parameters and the relative abundance of sulfolane degraders. Additionally, we investigated a fully-operational domestic GAC filtration system for the presence and abundance of known sulfolane degraders, which could indicate the potential for active biodegradation. We hypothesized that air sparging the aquifer removes sulfolane by stimulating aerobic biodegradation and predicted that the abundance of known sulfolane-degrading organisms would be elevated in that region as a result of overcoming the oxygen limitation on aerobic degradation in the subsurface.

## Materials and Methods

### Sampling

#### Site Description

**Figure 1:**
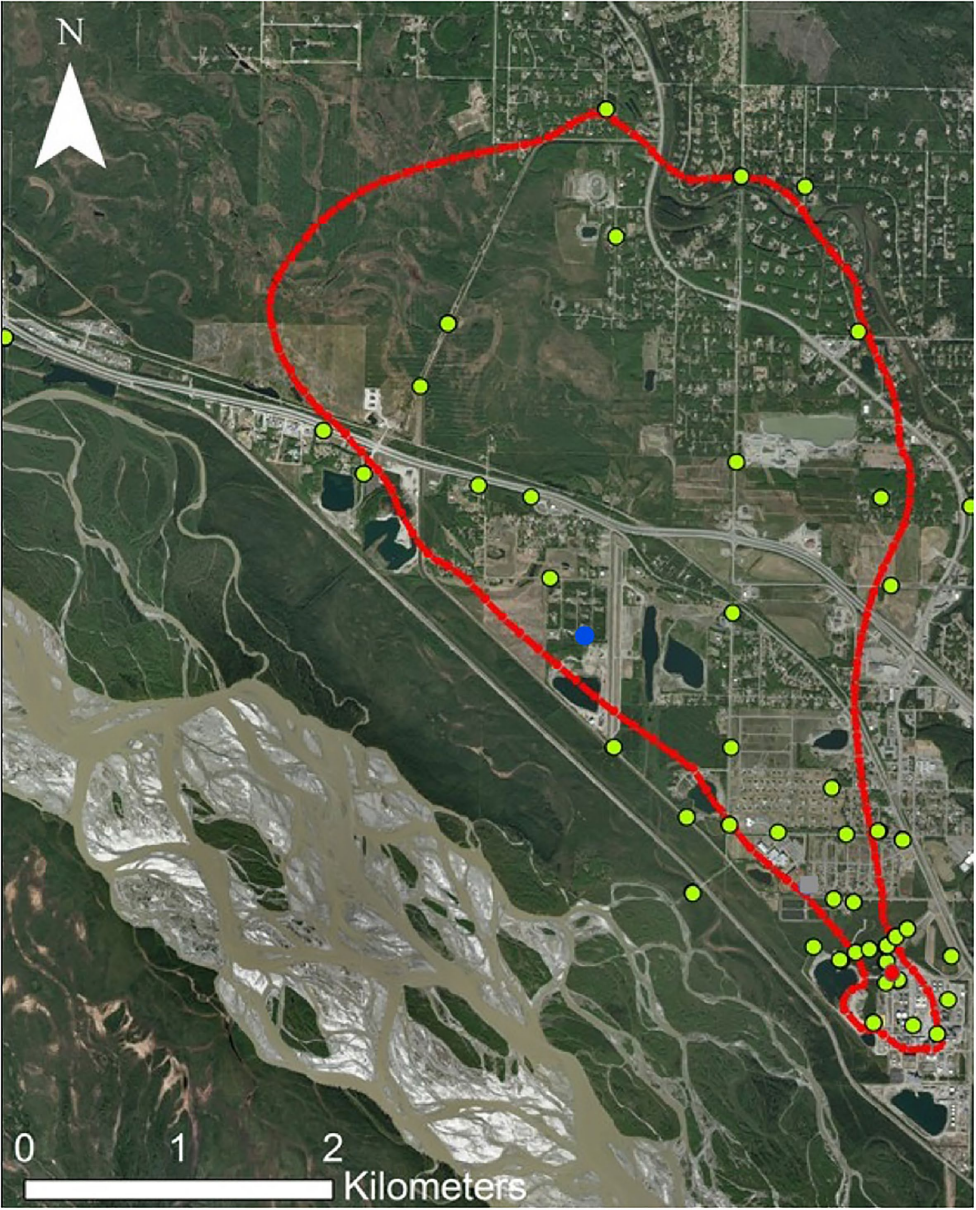
Map of the sulfolane plume in North Pole, Alaska. The red outline represents the extent of detectable sulfolane in the groundwater. The yellow dots represent monitoring wells sampled. The red dot represents the location of the experimental air-sparging system. The blue dot is the location of the granular activated carbon (GAC) system.

This study focuses on a sulfolane-contaminated groundwater plume located in North Pole, Alaska, United States (64.7511° N, 147.3519° W), which is part of the greater Tanana River aquifer supplied by the Alaska Range (Figure 1). This is an alluvial aquifer containing discontinuous permafrost. The sulfolane plume originated at a petroleum refinery where sulfolane use began in 1985 and ceased in 2014 when refinery operations ended. The size of the plume is estimated to be 5.6km long, 3.2km wide, and 91.4m deep and it continues to migrate to the north-northwest (Magdziuk and Andresen, 2018). Sulfolane concentrations throughout the plume ranged from below detection limits to 34.8mgL^−1^ at the time of sampling (Kurapati et al., 2014).

#### Plume-Wide Sampling

Groundwater samples were collected from MW installed throughout the contaminated groundwater plume. One hundred groundwater samples were collected from two routine sampling campaigns by environmental consultants Shannon and Wilson, Inc., under contract from refinery owners Flint Hills Resources. Eighty-two samples were collected between October 2nd and December 20th, 2013, and 18 samples were collected between January 7th and March 27th, 2014. Wells consisted of 5-cm diameter pipe with 0.05-cm screens, which were between 1.2 and 1.5m in length with half of the screen above and half below the targeted depth. Well depths ranged from 4 to 46m below ground surface. Prior to sample collection, groundwater was purged until geochemical parameters stabilized or three well volumes of groundwater were pumped from the well. A YSI ProPlus multiprobe or equivalent was used to monitor geochemical parameters, including temperature, conductivity, dissolved oxygen (DO), pH, and oxidation-reduction potential (ORP). One liter of groundwater was collected from each well and stored in a sterile 1-L bottle at 4°C until being filtered through a sterile 0.22-μm filter within 24h for microbial analysis. The filter was placed in a clean tube and stored at −80°C until DNA was extracted. A separate liter of groundwater was submitted to SGS Laboratories of Anchorage, Alaska, for sulfolane analysis using isotope dilution gas chromatography/mass spectrometry following a modification of USEPA Method 1625B.

#### Air Sparge System

The air sparge (AS) system pumped atmospheric air into the aquifer through eight injection wells with a 0.6-m well screen positioned 6.1m below ground surface in brown/gray gravely sand to sandy gravel soil with trace amounts of silt (Angermann and DeJournett, 2013). The air flow rate at each air sparge point was slightly variable and ranged from 42.5 to 76.5m^3^ h^−1^. Eight monitoring wells like those described above but surrounding the experimental AS system were also sampled for microbial community analysis in the same manner described above. One well was placed ~12m upgradient of general aquifer flow, three wells were down the center line of the system, two wells were ~4.5m outside the system to the west, and two wells were ~4.5m outside the system to the east. A schematic diagram of the AS system is provided in Supplementary Materials (Supplementary Figure 1).

When samples were collected from the AS wells sulfolane, temperature, conductivity, DO, pH, and ORP were analyzed as described above for MW samples. In addition, more detailed biochemical data were collected for the experimental air-sparge system, including dissolved iron, dissolved manganese, total organic carbon, and total phosphorus (Angermann and DeJournett, 2013). The air sparge system was started on March 7th, 2012 and was shut down on July 10, 2013 after 70weeks of operation (Kurapati et al., 2014). We obtained samples from 10, 13, and 70weeks after startup but were unable to acquire samples prior to system initiation due to limited access to the refinery.

#### Granular Activated Carbon From Point-of-Entry Treatment Systems

We examined the microbial community structure in a point of entry (POE) treatment system where GAC was used to sorb sulfolane from private wells (Supplementary Figure 2; BARR, 2011). On May 15th, 2014, a GAC canister from a POE system was received for microbial analysis. The canister had treated 78,160L of sulfolane-contaminated water prior to replacement. The canister was divided into thirds (top, middle, and bottom), and two 500-g samples were taken from each section for microbial community analysis (Janda, 2016). The GAC samples were stored at −80°C until DNA was extracted.

### DNA Extraction

DNA was extracted from the groundwater filters using a phenol-chloroform extraction method described in Miller et al. (1999) as modified by Hazen et al. (2010). For GAC samples, DNA was isolated using a MoBio PowerMax Soil DNA Isolation Kit following the manufacturer’s instructions. GAC samples were extracted in duplicate and pooled for sequencing. All DNA extracts were stored at −20°C prior to sequencing.

### Microbial Community Analyses

The bacterial and archaeal community structure was assessed in MW, GAC, and AS samples by sequencing a~250-bp segment of the V4 region of the 16S rRNA gene using an Illumina MiSeq as described in Kasanke et al. (2019). Amplicons were sequenced at the Michigan State University Research Technology Support Facility. FastQ files were analyzed using mothur software (1.35.1) following a modified version of the standard MiSeq SOP (accessed March 2016; Schloss et al., 2009; Kozich et al., 2013) as described by Martinez-Cruz et al. (2017). All sequences had a quality score of 25 or greater and the maximum contig length of 275. All unique sequences were aligned against the SILVA SEED v132 database, and chimera checking was performed using the mothur implementation of Uchime (Kozich et al., 2013; Quast et al., 2013). Unique operational taxonomic units (OTUs) were defined at a level of 99% sequence similarity and taxonomy was assigned using SILVA SEED v132 taxonomy database (Quast et al., 2013). BLASTN was used to obtain higher-resolution taxonomic assignment for dominant community members (Mount, 2007). To account for differences in sequence coverage, the number of sequences was subsampled to the number of sequences in the least covered sample after quality control steps (8,142). Samples were analyzed along with a larger dataset including a previously reported DNA-SIP experiment, where ^13^C-labeled sulfolane was added to North Pole aquifer substrate identifying a *Rhodoferax* sp. as the dominant sulfolane assimilating microorganism (Kasanke et al., 2019), allowing for direct OTU comparison of the labeled sulfolane-assimilating species. Sequence files used in this analysis are publicly available on the sequence read archive (SRA) under accession #PRJNA504308.

### Statistical Analyses

All multivariate statistical analyses were conducted using PCORD Version 6 statistical analysis software (McCune and Mefford, 2011). Differences between microbial communities were assessed using nonparametric Multi-Response Permutation Procedures (MRPP) from a rank-transformed Bray-Curtis distance matrix (McCune and Grace, 2002). To help identify species differences between the MW, AS, and GAC microbial communities, indicator species analysis was performed. Indicator values were calculated using the method of Dufrêne and Legendre (1997), and the significance of the indicator value was determined using 4,999 randomized Monte Carlo simulations. Community data were visualized using nonmetric multidimensional scaling (NMS) based on a Bray-Curtis distance matrix and random starting configurations with dimensionality of the data determined by comparison of 250 runs with real data and 250 randomized Monte Carlo simulations (Kruskal, 1964; Mather, 1976). Pearson and Kendall correlations of environmental variables with ordination axes were performed and variables with *R*^2^ values of 0.2 or greater were reported. Linear regressions were performed between all measured environmental variables (described above) and the abundance of known sulfolane degraders in the MW and AS systems, independently. *R* statistical software was used to conduct Welch’s two-sample *t*-tests, one-way ANOVA tests, *post-hoc* Tukey tests, and simple linear regressions (R Core Team, 2015). For all analyses, a value of *p* or an equivalent of 0.05 or less was considered significant. All reported values are the mean±SD.

## Results and Discussion

### Distribution of Sulfolane-Assimilating Species Throughout Plume and Treatment Systems

The primary goals of this study were to assess the abundance of known sulfolane-metabolizing bacteria throughout a contaminated aquifer and two groundwater treatment systems and to investigate environmental factors controlling their relative abundance. One treatment system, we examined is presumed to sorb sulfolane from private wells prior to human consumption using GAC (BARR, 2011). The other was an experimental AS treatment system, which successfully lowered sulfolane concentrations to below detection limits (6.88μgL^−1^) in the aquifer in downgradient test wells after 4–15weeks of operation (Angermann and DeJournett, 2013). While we found significant differences in overall microbial community composition between the AS, GAC, and MW samples (MRPP, significance of delta<<0.001, *A*=0.20), the initial focus was on the distribution of a *Rhodoferax* sp. identified as the dominant, if not exclusive, sulfolane-assimilating species in a DNA-SIP study that used substrate from this aquifer as inoculum^13^. We also examined the AS, GAC, and MW samples for the distribution of other known sulfolane-degrading microorganisms reported in the literature.

#### Plume-Wide Abundance and Environmental Drivers of *Rhodoferax* sp. Distribution

In our plume-wide survey of aquifer MW, we detected a total of 253 OTUs that were classified as members of the genus *Rhodoferax*, including the third most abundant groundwater bacterium detected. By re-analyzing our previous sulfolane SIP dataset along with the MW, AS, and GAC samples, we identified one of these OTUs as the previously described sulfolane-assimilating *Rhodoferax* sp. (Kasanke et al., 2019). We detected this known sulfolane-assimilating *Rhodoferax* sp. in 70% of the MW samples, suggesting that sulfolane biodegradation potential is widely distributed throughout the aquifer. However, the *Rhodoferax* sp. was generally present in relatively low abundance (maximum relative abundance of 4.1%; average 0.59±0.77%). The *Rhodoferax* sp. was detected in MWs without sulfolane (outside the plume) and *vice versa*, suggesting that its presence is not dependent upon the presence of sulfolane.

Although the *Rhodoferax* was present throughout the aquifer, it is unlikely that this organism actively degrades sulfolane under normal aquifer conditions. Prior incubation studies demonstrated that sulfolane biodegradation occurred only in this aquifer sediment and water under aerobic conditions (Kasanke and Leigh, 2017). However, thisaquifer is generally suboxic (Kurapati et al., 2014) and the monitoring well oxygen values at the time of sampling supported this (Median=7.5μM, Mean=14.3μM oxygen). Although there are reports of anaerobic sulfolane biodegradation at other sites (Greene et al., 1998; Kim et al., 1999), prior tests of anaerobic sulfolane biodegradation using substrate from this aquifer as the inoculum failed to result in sulfolane loss despite incubating for over 1,000days (Kasanke and Leigh, 2017). The consistently large size of the plume over time (5.6km long, 3.2km wide, and over 90m deep), with some continued expansion into previously uncontaminated wells observed (Kurapati et al., 2014; Magdziuk and Andresen, 2018), provides further evidence that appreciable degradation of sulfolane is not occurring under ambient aquifer conditions, despite the presence of the sulfolane degrader.

**Figure 2:**
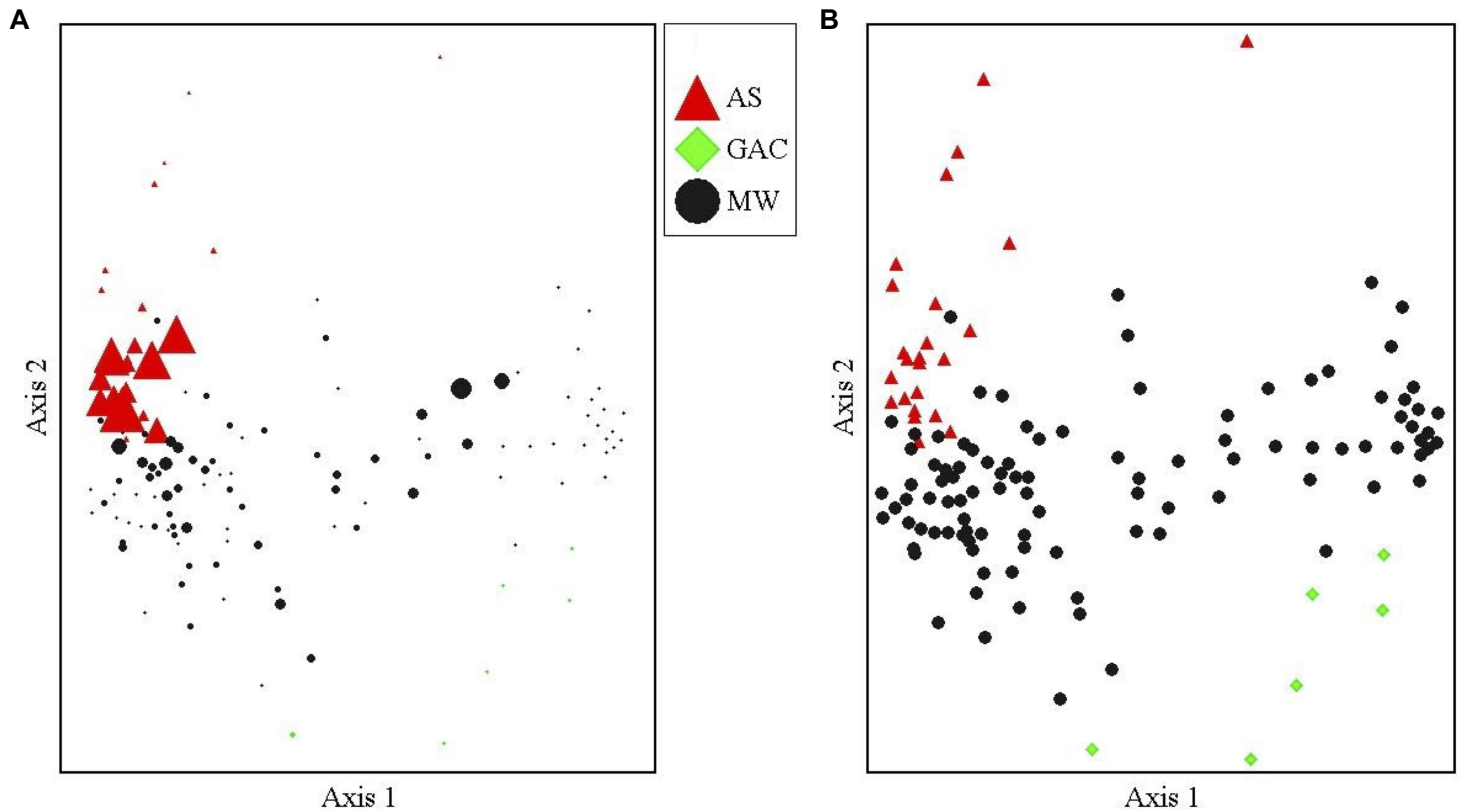
NMDS of the aquifer monitoring well (MW), air sparge system (AS), and GAC microbial communities. **(A)** Marker size is proportional to the relative abundance of the sulfolane-assimilating *Rhodoferax* sp. in each sample. Largest size marker represents a 10.5% relative abundance and smallest represents 0% relative abundance. **(B)** Identical nonmetric multidimensional scaling (NMS) plot with equal marker sizes for each sample showing the microbial community group by sample type. A stable solution was reached after 58 iterations with an optimal dimensionality of two axes, a final stress of 18.6 and a final instability value of 0.00000. The proportion of variance explained by each axis was 36.7% for axis 1 and 16.9% for axis 2.

Oxygen limitation appears to be inhibiting the growth and activity of the sulfolane-assimilating *Rhodoferax* sp. in this aquifer. We found that the AS treatment system fostered significantly higher abundances of the sulfolane-assimilating *Rhodoferax* sp. than the surrounding aquifer (Welches two-sample *t*-test, df=23.6, *t*=4.66, *p*=0.0001; Figure 2), with this bacterium being the most abundant OTU in the AS samples (maximum relative abundance 10.5%; average 3.6±3.2%). Indicator species analysis found the sulfolane-assimilating *Rhodoferax* sp. to be a strong indicator of the AS samples when compared to MW and GAC samples with an indicator value of 80.9 (*p*=0.0002; Supplementary Table 1). The dominance of the known sulfolane degrader in this system combined with the reduction in sulfolane concentration (Supplementary Table 2; Angermann and DeJournett, 2013) suggest that the AS system stimulated sulfolane biodegradation *in situ*. This is consistent with previous microcosm studies involving air sparging of sulfolane-contaminated soil, which attributed sulfolane loss exclusively to aerobic biodegradation (Greene and Fedorak, 2001). Sulfolane has a low vapor pressure (Sander, 2015) and strong affinity for water (Ashcroft et al., 1979), so air sparging cannot remove the contaminant through volatilization (Huang and Shang, 2006). There was no evidence to suggest a role for abiotic degradation of sulfolane in the AS system, as abiotic sulfolane losses have never been observed in sterile controls among all sulfolane biodegradation studies published to date (Greene et al., 1998, 2000; Kasanke and Leigh, 2017). All prior reports of abiotic sulfolane degradation involved the use of strong oxidizers, radiation, or both (Agatonovic and Vaisman, 2005; Izadifard et al., 2017).

**Figure 3:**
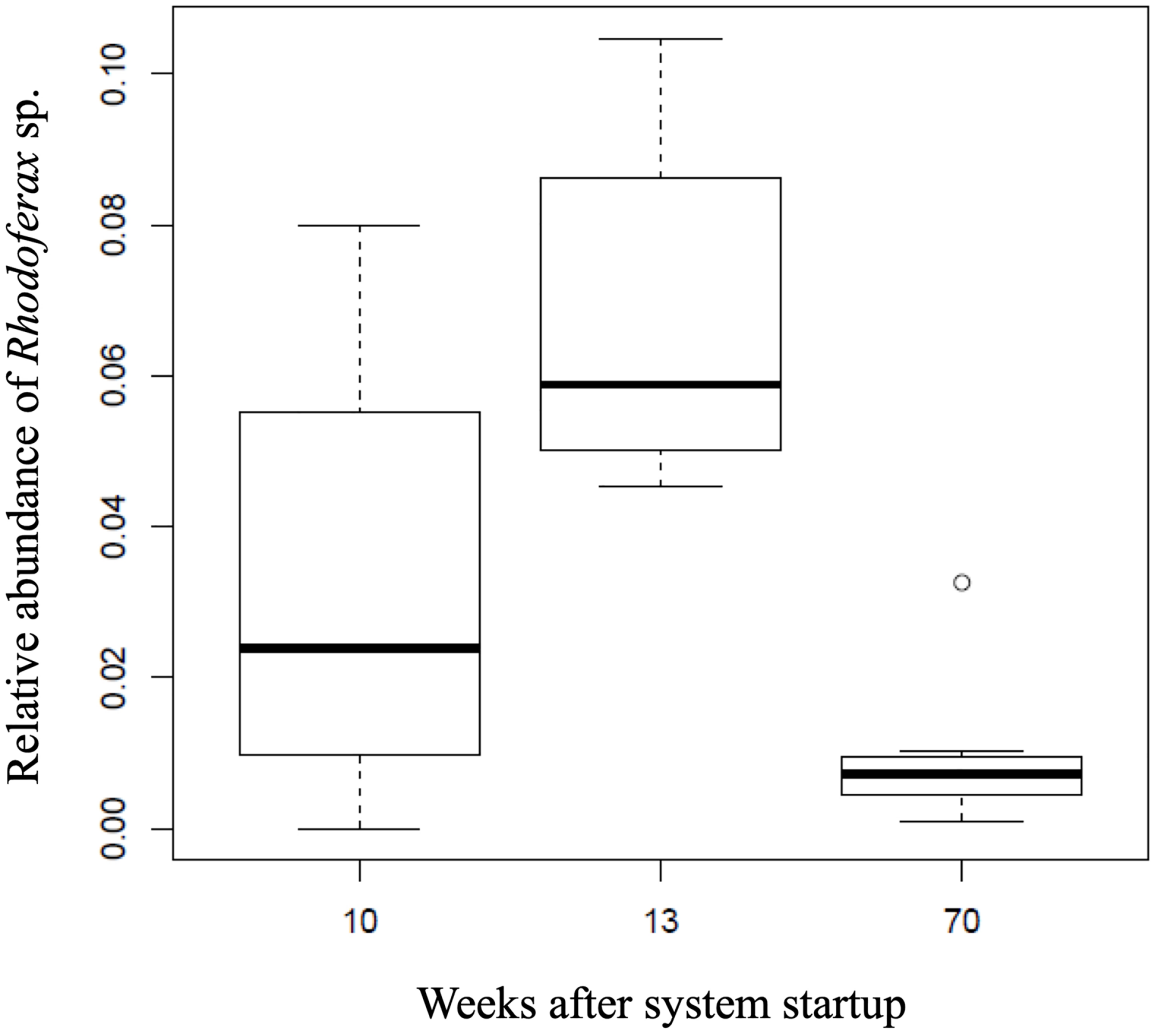
Relative abundance of the sulfolane-assimilating *Rhodoferax* sp. in groundwater samples collected from the AS system after 10, 13, and 70 weeks of operation.

Temporal shifts in the relative abundance of the *Rhodoferax* sp. were detected in AS communities with respect to the amount of time the AS system was active (ANOVA, *F*_2,21_=14.96, *p*<0.001). The DO concentrations in downgradient AS wells ranged between 0.49 and 17.56mgL^−1^ with an average of 9.77±5.51mgL^−1^. Initial sulfolane concentrations in the AS system prior to activation ranged from 71.7 to 278μgL^−1^ and dropped to below detection limits (6.88μgL^−1^) in downgradient test wells after 4–15weeks of operation (Angermann and DeJournett, 2013; Supplementary Table 2). As the sulfolane concentration decreased in the AS system, the sulfolane-assimilating *Rhodoferax* sp. increased in abundance from week 10 to 13 (*p*<0.009), suggesting biodegradation as a mechanism of sulfolane loss in this system (Figure 3). On week 70, the monitoring well upgradient of the AS system still had measurable levels of sulfolane, indicating a constant feed of new sulfolane into the system throughout the duration of the experiment (Supplementary Table 2). After 70weeks of air sparge operation, the relative abundance of the *Rhodoferax* sp. was significantly reduced (*p*<0.001; Figure 3). This decrease may be a result of community resilience after contaminant exposure ceases (Boivin et al., 2006) since sulfolane was not detected downgradient of the AS system after 15weeks of operation (Angermann and DeJournett, 2013). The decrease in *Rhodoferax* sp. abundance could also result from a depletion of specific nutrients necessary to support the growth of this strain (Bren et al., 2013), which can occur with prolonged biodegradation. Finally, oxygen addition can also stimulate the growth of bacterial grazers in the air-sparge system, which has been shown to influence bacterial population dynamics in groundwater (Nagaosa et al., 2008).

**Figure 4:**
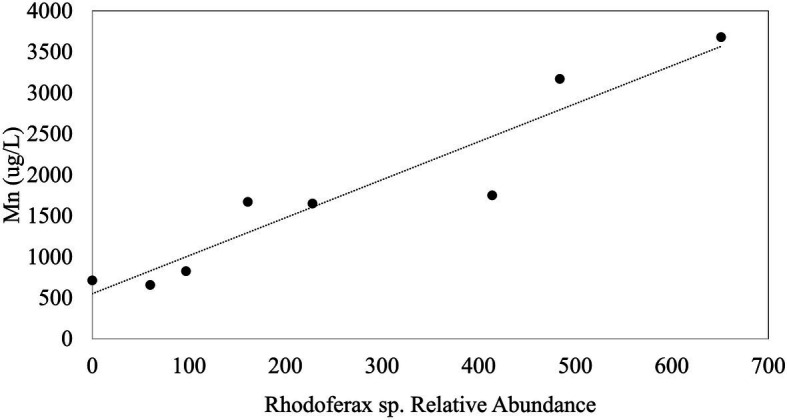
Plot of the linear regression between the relative abundance of the sulfolane degrading species and Mn during week 10 of AS system operation (*p*<0.001; *R*^2^=0.88).

In order to explore environmental controls on sulfolane biodegradation potential throughout the plume, we examined biogeochemical properties of groundwater obtained from MWs in relation to the relative abundance of the *Rhodoferax* strain. The environmental variables we measured (i.e., sulfolane, temperature, conductivity, DO, pH, and ORP) did not explain variations in the relative abundance of the sulfolane-assimilating *Rhodoferax* sp. throughout the aquifer MWs. Although statistically significant correlations of this species with temperature and sulfolane concentration were detected, the goodness of fit values were extremely low (*R*^2^=0.065 and 0.061, respectively) indicating these variables are not reliable predictors of the distribution of this species. Although there were generally higher abundances of the *Rhodoferax* in the AS system than elsewhere in the plume, we also found no correlations with the environmental variables collected for the AS system when combining the data from weeks 10, 13, and 70 of system operation. During week 10 of AS system operation, there was a significant (*p*<0.001) and strong (R^2^=0.88) positive correlation between dissolved Mn concentration and the relative abundance of the *Rhodoferax* sp. (Figure 4). This correlation did not exist at weeks 13 and 70 of AS system operation, raising some uncertainties as to whether this is a spurious observation. Nonetheless, the findings suggest that the availability of Mn may play a role in either stimulating or limiting the growth of this sulfolane-assimilating bacterium. Mn is known to be used by bacteria as a cofactor in superoxide dismutase enzymes which are important in defending against oxygen toxicity by catalyzing the transformation of oxygen radicals into water (Martin et al., 1986; Miller, 2012). These manganese-containing enzymes have been shown to be upregulated by bacteria in the presence of oxygen (Amo et al., 2003). Although speculative, it is possible that the positive association between the *Rhodoferax* sp. and Mn is related to the ability to produce enzymes that counteract oxygen toxicity. We were unable to measure dissolved Mn concentrations in the MW samples. Future attempts to determine environmental controls on the distribution of sulfolane degrading microorganisms should include a more thorough elemental analysis, including measuring dissolved Mn concentrations, as well as other environmental factors with the potential to influence microbial activity. The lack of correlation between abundance of the *Rhodoferax* sp. and DO in the AS system was surprising, but might be explained by the decline in the *Rhodoferax*’s relative abundance over time as sulfolane or nutrients became depleted in the AS zone.

#### Role of Sulfolane Degraders in Granular Activated Carbon Water Treatment Systems

In the GAC treatment system, the sulfolane-assimilating *Rhodoferax* sp. was only detected in half of the samples (relative abundance of 0.37, 0.11, and 0.025%). The obligate anaerobe *Ferribacterium limneticum* (99% identical *E* value=2e-128) was the most dominant bacterium representing 38.6±19.2% of the GAC community suggesting that the GAC system was primarily anoxic (Cummings et al., 1999). Although thermodynamically feasible, anaerobic sulfolane biodegradation has only been reliably observed in 4 of 60 anaerobic microcosms inoculated with sediment from Western Canada (Greene et al., 1998). Prior anaerobic incubations conducted using aquifer substrate from North Pole, Alaska, under nitrate-, sulfate-, and iron-reducing conditions resulted in no sulfolane loss after 1,021days of incubation (Kasanke and Leigh, 2017). GAC column sorption studies conducted prior to home installation of GAC POE systems in North Pole showed predictable sulfolane breakthrough curves consistent with sorption models (BARR, 2011). Additional studies support that sulfolane is readily sorbed by GAC (Diaz, 2015). All these findings support the conclusion that sorption, not biodegradation, is the mechanism of sulfolane removal in the North Pole GAC systems and that routine GAC changeout prior to sulfolane saturation and breakthrough remains an important part of their safe management.

This conclusion is in contradiction with previous research that found it necessary to inoculate GAC with microorganisms obtained from sewage plant effluent to remediate sulfolane (Ying et al., 1994). However, the sewage plant research was done in the presence of the co-contaminants dibromochloropropane and ethylene dibromide, which preferentially sorb to GAC. In the absence of co-contaminants or other organics that compete for sorption sites on GAC, it does not appear that biodegradation is a necessary component of GAC treatment in order to achieve removal of sulfolane from contaminated water. Our investigation only examined one GAC system, however, and other GAC-based systems may function differently and involve a combination of biotic and abiotic removal mechanisms.

#### Distribution of Other Known Sulfolane Degraders in the Plume and Treatment Systems

In addition to screening for the sulfolane-metabolizing *Rhodoferax* strain found in our earlier study of this aquifer, we also queried our MW, AS, and GAC datasets for sulfolane degraders previously reported by others in different geographic regions. To our knowledge, only four other sulfolane degrading microorganisms have been isolated from the environment. *Pseudomonas maltophilia* was isolated from the soil of an abandoned strip mine near Cambria, Illinois (Lee and Clark, 1993); a novel *Shinella* sp. was isolated from soil in the Yambaru area of Okinaw a Main Island; Japan (Matsui et al., 2009), and a strain of *Cupriavidus plantarum* was isolated form a petrochemical wastewater treatment plant in Taiwan (Yang et al., 2019). One OTU in our dataset was classified as a *Cupriavidus* sp. that was 99% identical (*E*-value=4e-131) to the known degrader. That OTU was only detected in one MW sample indicating that it is not widespread, although it did represent a relatively high 1.9% of the community in that sample. We did not detect any *Shinella* spp. in the dataset and, among the 36 *Pseudomonas* spp. detected, none were matches to *Pseudomonas maltophilia*. In western Canada, a sulfolane-degrading *Variovorax* sp. described as being closely related to *Variovorax paridoxus* was isolated from sulfolane-contaminated aquifer substrate (Greene et al., 2000). A *Variovorax* sp. 99% identical to *Variovorax paradoxus* (*E*-value=2e-128) represented 86.1% of the community in one AS well only in week 10. Interestingly, this single sample was the only AS sample in which the sulfolane-assimilating *Rhodoferax* sp. was not detected. The elevated abundance of this species in a portion of the AS system relative to the MW samples (max relative abundance 0.04%) and close relation to a known sulfolane degrader suggests this bacterium also may have been degrading sulfolane. By week 13, a community shift occurred, and this AS well also became dominated by the sulfolane-assimilating *Rhodoferax* sp.

## Conclusion

We characterized the microbial communities associated with a sulfolane-contaminated aquifer and two sulfolane treatment systems with special attention to the distribution of known sulfolane-degrading microorganisms to understand the environmental controls on the potential for sulfolane biodegradation and bioremediation. Biodegradation potential was widely distributed throughout the contaminated aquifer, as evidenced by the presence of the aerobic sulfolane-degrading *Rhodoferax* strain in low abundance in over 70% of samples. However, the size and persistence of the sulfolane plume, combined with prior laboratory evidence of biodegradation only under aerobic conditions, suggest that no appreciable intrinsic sulfolane biodegradation is occurring under the prevalent suboxic conditions in the plume. Air sparging effectively reduced sulfolane levels concomitantly with an increase in the relative abundance of a *Rhodoferax* sp. known to metabolize sulfolane under aerobic conditions, suggesting that biostimulation of aerobic biodegradation may be the mechanism for sulfolane removal. Biodegradation was not suspected to be a mechanism underlying sulfolane removal from contaminated water using aboveground GAC treatment, based on the very low abundance of the degrader and prior evidence that GAC readily sorbs sulfolane. Manganese was the only environmental variable that correlated with the relative abundance of the sulfolane degrader and warrants further investigation for its relationship to the sulfolane-degrading *Rhodoferax* sp. Overall, we provide novel insights into the basic microbial ecology of a subarctic aquifer and the fundamental mechanisms behind effective sulfolane remediation systems that may be valuable to assessing and remediating other sulfolane-contaminated sites.

## Data Availability Statement

The datasets presented in this study can be found in online repositories. The names of the repository/repositories and accession number(s) can be found at: https://www.ncbi.nlm.nih.gov/, PRJNA504308.

## Author Contributions

CK, MW, and MBL contributed to conception and design of the study and wrote sections of the manuscript. MW and CK collected and processed samples and created figures and tables. CK performed the statistical analysis and wrote the first draft of the manuscript. All authors contributed to the article and approved the submitted version.

## Conflict of Interest

The authors declare that the research was conducted in the absence of any commercial or financial relationships that could be construed as a potential conflict of interest.

## Publisher’s Note

All claims expressed in this article are solely those of the authors and do not necessarily represent those of their affiliated organizations, or those of the publisher, the editors and the reviewers. Any product that may be evaluated in this article, or claim that may be made by its manufacturer, is not guaranteed or endorsed by the publisher.

## Supplementary Material

Supplementary Table 1 Indicator species table containing OTUs with an indicator value (IV) >70 and a value of *p* <0.05.

Supplementary Table 2 Sulfolane concentration (μg/L) over time in air-sparge treatment system monitoring wells.

Supplementary Figure 1 Layout of the experimental air-sparge system.

Supplementary Figure 2 Schematic of the granular activated carbon remediation system.
