# Supplementary figures and images for: Distribution of a Sulfolane-Metabolizing Rhodoferax sp. Throughout a Contaminated Subarctic Aquifer and Two Groundwater Treatment Systems
Source: Front Microbiol. 2021 Aug 26;12:714769. doi: 10.3389/fmicb.2021.714769 (PMC8427821; doi:10.3389/fmicb.2021.714769)

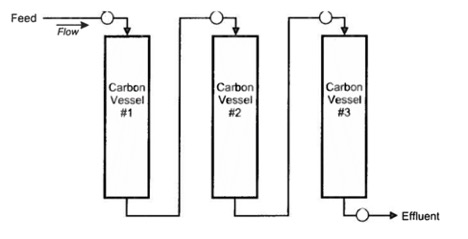

Supplement: Supplementary Figure 2 — Schematic of the granular activated carbon remediation system. [file Image_2.JPEG]
