# Supplementary material for: Distribution of a Sulfolane-Metabolizing Rhodoferax sp. Throughout a Contaminated Subarctic Aquifer and Two Groundwater Treatment Systems
Source: Front Microbiol. 2021 Aug 26;12:714769. doi: 10.3389/fmicb.2021.714769 (PMC8427821; doi:10.3389/fmicb.2021.714769)
Supplement: Supplementary Figure 1 — Layout of the experimental air-sparge system. [file Image_1.PDF]

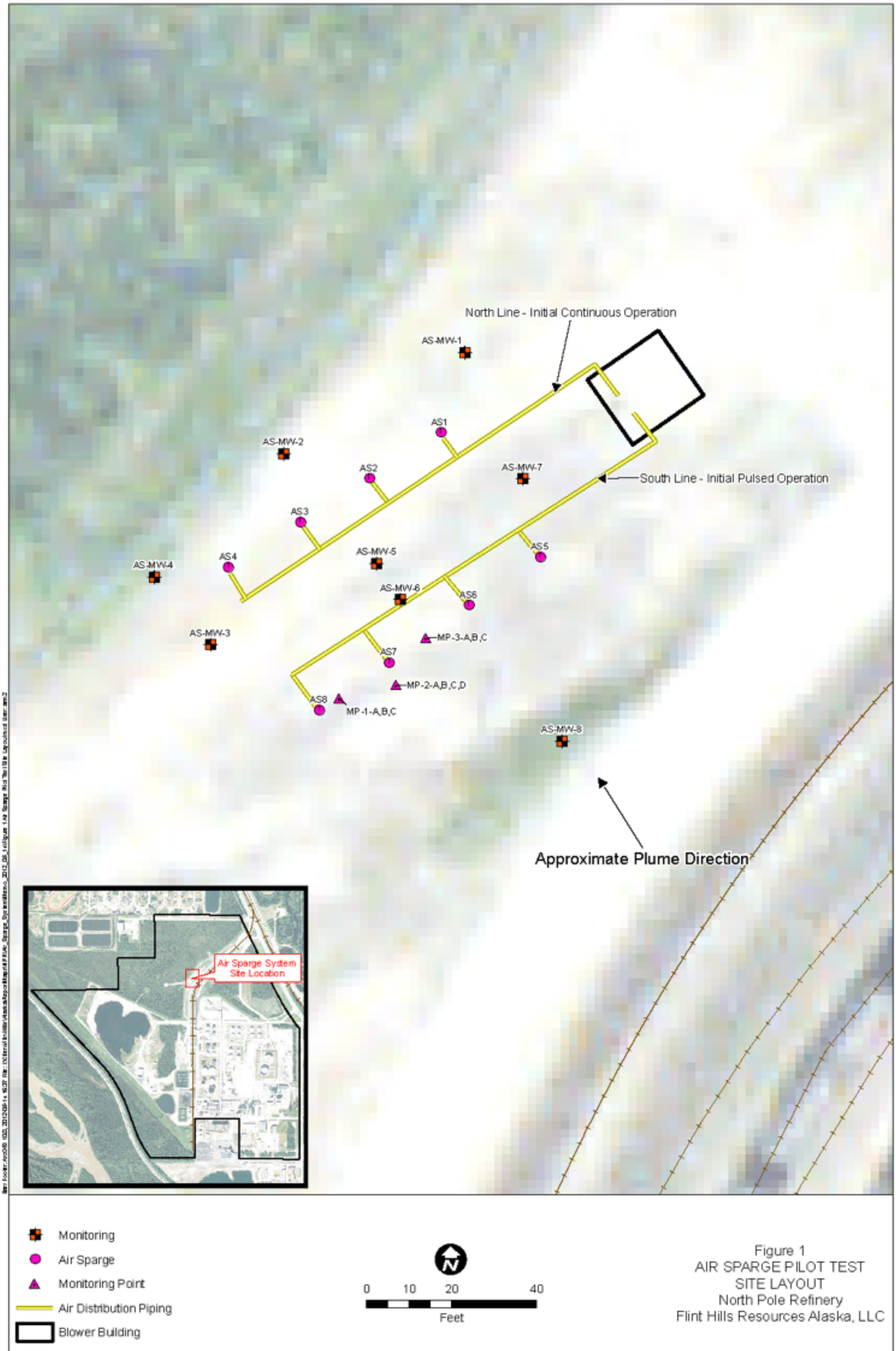

Figure 1
AIR SPARGE PILOT TEST
SITE LAYOUT
North Pole Refinery
Flint Hills Resources Alaska, LLC
